# Ovarian ectopic pregnancy: A 10 years’ experience and review of literature

**Published:** 2014-12

**Authors:** Lajya Devi Goyal, Rimpy Tondon, Poonam Goel, Alka Sehgal

**Affiliations:** 1 *Department of Obstetrics and Gynecology, Guru Gobind Singh Medical College and Hospital, Faridkot, Panjab, India. *; 2 *Department of Obstetrics and Gynecology, Govt. Medical College, Chandigarh, India.*

**Keywords:** *Ectopic pregnancy*, *Ovarian pregnancy*, *Infertility*

## Abstract

**Background:**

Primary ovarian pregnancy is one of the rarest forms of ectopic pregnancy having incidence of 1/7000-1/40,000 in live births and 0.5-3% of all ectopic gestations. Intrauterine contraceptive device (IUCD), salpingitis, infertility, and assisted reproductive techniques are the important risk factors. Approximately, 75% terminate in first trimester and are often misdiagnosed as corpus luteum haemorrhage. Preoperative diagnosis by ultrasonography (USG) in early pregnancy can help in conservative medical/ surgical management.

**Objective:**

The aim of the present study was to find the incidence, risk factors, role of USG in pre-operative diagnosis, feasibility of conservative management with medical method or minimal invasive surgery in developing countries like India.

**Materials and Methods:**

We did a retrospective cross-sectional study of ovarian pregnancies managed at Government Medical College and Hospital Chandigarh between July 2000 to July 2010. We analyzed the incidence, risk factors, clinical presentation, management of ovarian pregnancy, and reviewed the literature.

**Results:**

Incidence of ovarian pregnancy was 4.9% of all ectopic pregnancies (14/523). Thirteen (93%) patients presented in first trimester with acute pain abdomen and of these ten patients had bleeding per vaginum. One (7%) patient referred from peripheral hospital at term gestation with ultrasonographic diagnosis of breech presention with plecenta previa. Pre-operative diagnosis was made only in two cases (11%). All cases were managed by laparotomy. Excision of the sac with conservation of the ovary was done in eleven cases (78%) and oophorectomy was done in two cases (14%).

**Conclusion:**

Incidence of ovarian pregnancy is on the rise. Although ultrasonography can detect ovarian gestations in unruptured cases but cannot easily differentiate ovarian from other tubal gestation in ruptured state. Medical management is usually not feasible it most of the patients present in ruptured state. Conservative surgical approach is the management of choice.

## Introduction

Ovarian pregnancy is uncommon form of ectopic pregnancy with an incidence of 1/7000-1/40,000 live births and 0.5-3% of all ectopic gestations (1). Advance ovarian pregnancies are exceptional. Approximately 75% terminate in first trimester and are often misdiagnosed as corpus luteum haemorrhage (2). The Spielberg criteria (1878)- a) fallopian tube as the affected site must be intact b) the foetal sac must occupy the position of the ovary c) the ovary must be connected to the uterus by ovarian ligament and d) ovarian tissue must be located in the sac wall, are essential for confirmation of early ovarian pregnancy. In advance pregnancies last criterion i.e. detection of ovarian tissue in the wall of sac may not be satisfied as parenchyma is compressed laminated and distended by developing foetus (3). 

The actual incidence could be up to 1 in 1400 deliveries if the criteria other than those of Spielberg are taken into consideration. These criteria combine biochemical and USG findings and include a) serum βhCG level ≥1000 IU/L b) no gestational sac in uterine at transvaginal ultrasound c) ovarian involvement should be confirmed on exploration, with bleeding, visualization of chorionic villi or presence of atypical cyst as the ovary d) normal tubes e) absence of serum βhCG after treatment of ovary (4). Intrauterine contraceptive device (IUCD) use was the only risk factor quoted in the past literature and salpingitis and infertility are not implicated but recent literature quote increased incidence with infertility and assisted reproductive techniques (5-7). 

Role of USG in diagnosis of ovarian gestation has been described but most of the patients present with ruptured ectopic and are in circulatory collapse so preoperative diagnosis of ovarian ectopic on sonography is not easy (8, 9). We conducted a retrospective analysis and reviewed the case records of fourteen ovarian pregnancies including one case of term ovarian pregnancy among 523 ectopic gestations and 33285 deliveries. 

The aim of the present study was to find the incidence, risk factors, possibility of pre-operative USG diagnosis, feasibility of conservative management with medical method or minimal invasive surgery. 

## Materials and methods

A retrospective cross-sectional study of ovarian pregnancies was conducted in department of Obstetrics and Gynecology in our Institute over a period of ten years i.e. July 2000-July 2010. The case records of ovarian pregnancies were searched in central record department of the hospital after taking permission from medical record section to review the records of all those patients diagnosed with ovarian pregnancy for academic and research purpose. 

Verbal consent was taken telephonically from all the patients. Baseline information, presenting features, investigations and operative details were recorded. There is no control group in this study, because it investigates and reports the present files.

## Results

There were 523 ectopic pregnancies among 33285 deliveries (1.7%). In ectopic gestations fourteen were ovarian, four cervical, three abdominal and 502 were tubal gestation. All patients fulfilled Speigelberg’s criteria. One case of ovarian pregnancy presented at term (10). The mean age of the patients was 27 years. Mean gestational age was 42 days. Eight patients presented with amenorrhea. One patient presented at term gestation and in five patients there was no history of amenorrhea. Most common presenting complaint was acute pain abdomen in 13 (93%) patients followed by vaginal bleeding in 10 (71%) patients. Six patients showed evidence of circulatory collapse at presentation. Vaginal findings showed ectopic pregnancy in eleven patients. There was history of IUCD use in five cases (duration 1-6 years). 

History of infertility was present in two cases, of these one patient had undergone ovulation induction and intra uterine insemination in the preceding cycle. History of spontaneous or induced abortion was present in five cases and six patients were nullipara (three were primigravida and three had previous one or two first trimester abortions). Pregnancy test was positive in thirteen patients (pregnancy test was not done in case of term ovarian pregnancy). βhCG test was done in seven patients only (range: 1600-3200 IU/L) as other patients could not afford due to financial constraints. 

USG findings revealed echogenic sac with fetal cardiac activity in the ovary only in two cases. Adnexal mass was present on USG in five cases varying from 4x4cm to 7x6cm. Rest of the cases showed free fluid in peritoneal cavity with clots in pouch of doughlas. Term ovarian pregnancy was misdiagnosed as breech presentation with placenta previa on USG. All patients underwent laparotomy. Intra operative findings revealed sac in the process of extrusion and surface bleeding from right ovary in nine cases. 

Term ovarian pregnancy was also in the right sided ovary. Left ovarian pregnancy presented as an adnexal mass in two cases and ruptured cystic mass in two cases. Intra operative diagnosis of corpus luteal hemorrhage was suspected in three cases however ovarian pregnancy were confirmed by histopathology examination in all these cases. Excision of the sac with conservation of the ovary was done in 11 cases. Oophrectomy was done in two cases as sac was deep seated and bleeding could not be controlled. In term ovarian pregnancy entire ovary was incorporated in sac so excision of the sac was done. On follow up pregnancy test was negative in all cases of early ovarian pregnancies (Table I).

**Table I:** Age, parity, risk factor, diagnosis and treatment of ovarian pregnancy

Serial number	Age (years)	Parity	Period of Gestation (weeks)	Risk Factors	Ultrasonography Findings/ β-hCG	Intraoperative findings	Treatment
1	24	G 5P2L2A2	6+2	CuT-2yr	Ecogenic Sac with FCA in R adnexa	sac extruding from R ovary	Excision of sac B/L salpingectomy
2	22	Nulliparous	Nil	Primary infertility ovulation induction, IUI	FF in peritoneal cavity	sac extruding from L ovary	Excision of sac
3	26	G4P2L2A1	6	CuT-1yr	sac in R ovary with FCA βhCG -2200iu/l	G sac in R ovary	Excision of sac B/L salpingectomy
4	28	P3L3	Nil	CuT-3yrs	5×5 cm L adnexal mass, echogenic area	L ovary embedded in clots	L Oophrectomy
5	24	G2P1L1	40	MTP in early pregnancy Continuation of pregnancy	Placenta previa, Breech, oligohydraminos	Live 2.2 kg fetus in R ovarian sac	Excision of sac
6	35	G2P1L1	6+1	Nil	FF in peritoneal cavity βhCG 1880iu/l	R ovarian 2cm mass	Excision of cyst
7	24	Primi	6+1	Nil	R adnexal mass FF	Protruding sac from R ovary	Excision of sac
8	35	G3P2L2	5	CuT-6yr	R ovarian sac with fetal node. βhCG 2400iu/l	R ovarian sac	Excision of sac
9	24	Primi	6+2	Nil	FF in abdomen	1.5 cm rent in R ovary	Excision of cyst
10	26	P3L3	Nil	Nil	L adnexal mass, βhCG -3200iu/l	5×5 cm L ovarian ruptured mass	Excision of cycst and repair
11	32	Nulliparous	Nil	Nil	L adnexal mass βhCG 1800iu/l	Ruptured sac in L Ovary	Excision of sac
12	26	G5P1L1A3	7+2	CuT-2yr	R adnexal mass, βhCG -3000iu/l	5×4cm R ovarian Cyst	Excision of sac
13	20	G2A1	5+2	Nil	FF in abdomen	Sac extruding R ovary	Excision of sac
14	32	Nulliparous	Nil	Infertility	R adnexal mass βhCG-2400iu/l	Sac in R ovary	R Oophrectomy

FCA:Fetal cardiac activity

R: Right

L: Left

FF: free fluid

B/L: bilateral

## Discussion

Although ovarian pregnancy is a rare entity but incidence is on rise. Quoted incidence in past were 0.5-1% of all ectopic gestation or 1 in 7,000-40,000 live births (1). In our series incidence of ovarian pregnancy were 1.7% of all ectopic and 1 in 3332 deliveries which almost was doubled. Recent studies suggest that the actual incidence could be up to 1 in 1400 deliveries if the criteria other than those of Speigelberg’s are taken into consideration (4). 

There is overall increase incidence of ectopic gestation due to increasing prevalence of sexually transmitted disease and PID, induced abortions, assistant reproductive techniques and increased availability of diagnostic facilities. Increased incidence of ovarian pregnancies may be associated with IUCD as it prevents intrauterine but not extra uterine pregnancies. It is postulated that IUCD may potentiate ovarian nidation due to changes in synthesis of prostaglandin which increase the tubal peristalsis that could increase incidence of both tubal and ovarian pregnancies (5). Five out of fourteen patients had history of CuT use in our series. Recent literature quote increased incidence with infertility and assisted reproductive techniques (7, 8).

Two patients in our series had history of primary infertility and one underwent intrauterine insemination in preceding cycle and five patients had previous history of induced abortions. Choi *et al* reported endometriosis and previous abdominal surgeries are the most common risk factors in his series of 49 cases of ovarian pregnancy but none of our patients had features of endometriosis or history of abdominal surgery (11). Recently ovarian ectopic pregnancy has been reported after tubal ligation (12).

Although the ovary can accommodate itself more readily than the tube to the expanding pregnancy but 75% of ovarian gestation rupture in the early first trimester and are usually misdiagnosed as corpus luteal haemorrhage (2). Advanced ovarian pregnancies are exceptional (in our series term ovarian pregnancy with live birth has been reported). Term healthy live birth out of ovarian pregnancy is very rare. Although advanced ovarian pregnancies with intrauterine death have been reported (3).

Classical triad of ectopic pregnancy i.e. amenorrhoea, pain abdomen and vaginal bleeding was present only in six cases. Role of USG in diagnosis of ovarian gestation has been described by various authors. The following sonographic diagnostic criteria for the presence of an ovarian pregnancy have been suggested: a wide echogenic ring with an internal echo lucent area on the ovarian surface; the presence of ovarian cortex, including corpus luteum or follicles around the mass; and the echogenicity of the ring usually greater than that of the ovary itself (8, 9). Recently, a case was diagnosed with 3-dimensional sonography (13). 

Preoperative diagnosis by transvaginal USG and βHCG should be attempted but it is possible only in patients who present early with pelvic pain and vaginal bleeding. Most of the patients present with ruptured ectopic and are in circulatory collapse so preoperative diagnosis of ovarian ectopic on sonography is not easily made. Ovarian tumour producing HCG also may mislead the accurate diagnosis. Intraoperative diagnosis could be made in only 28% in a series of 25 cases, because it was difficult to distinguish an ovarian pregnancy from a hemorrhagic corpus luteal cyst (2). 

Preoperative diagnosis was possible only in two cases in our series. All fourteen patients were confirmed by histopathology examination in our series. Ovarian pregnancy had been treated by ipsilateral oophorectomy in past, but the trend has been shifted toward conservative surgery such as cystectomy or wedge resection performed at either laparotomy or laparoscopy. Management options like medical therapy with methotrexate, laparoscopic assisted medical therapy with etoposide have been tried with various success rate (14-16).

Treatment of choice is resection of sac and hemostasis preferably with laparoscopy or laparotomy as most of the patients arrive in collapsed state with uncertain diagnosis and laparoscopy may not be feasible (17). In our series sac excision and hemostasis were performed in eleven cases as sacs were already ruptured with products of conception extruding in these patients. In two patients oopherectomy had to be resorted because of uncontrolled hemorrhage and hemostasis was not possible. 

In case of term ovarian pregnancy excision of placenta containing sac was performed. Although none of the patients became pregnant in one year follow up in our series but literature suggest there is a high rate of successful subsequent pregnancy and a low rate of subsequent ectopic pregnancy or of infertility (18).

## Conclusion

Incidence of ovarian pregnancy is on rise due to increased incidence of infertility and use of assisted reproductive techniques. Ultrasonography can detect ovarian gestations in unruptured cases but cannot easily differentiate ovarian from other tubal gestation in ruptured state. As most of the patients present with ruptured sac in collapsed state medical management is usually not feasible. Conservative surgical approach remains the management of choice.
